# What did the scientific literature learn from internal company documents in the pharmaceutical industry? A scoping review

**DOI:** 10.1002/cesm.12011

**Published:** 2023-04-27

**Authors:** Marc‐André Gagnon, Miaoran Dong

**Affiliations:** ^1^ School of Public Policy and Administration Carleton University Ottawa Ontario Canada; ^2^ School of Journalism and Communication Carleton University Ottawa Ontario Canada

## Abstract

**Objective:**

To identify all scientific papers that used internal industry documents in the pharmaceutical sector and analyze what and how the scientific literature learned about corporate influence in the pharmaceutical sector through these internal documents.

**Design:**

Scoping review.

**Methods:**

Using different series of keywords, we searched six databases, PubMed, Scopus, Web of Science, CINAHL, Business Source Complete, and PAIS, for peer‐reviewed journal articles analyzing pharmaceutical corporations' internal documents. We completed the scoping review using a purposive snowball sampling method to extract relevant case studies and peer‐reviewed journal articles from relevant articles' reference lists when our search keywords failed to capture them. To analyze the content of the literature and better categorize the types of corporate strategies at play in the pharmaceutical sector, we used categories of ghost‐management previously developed in the literature.

**Results:**

We identified 37 peer‐reviewed papers in the final results. All the articles included in the final results are published in English. Almost all articles obtained most of their internal document data through legal proceedings. All 37 articles unveil dynamic ghost‐management strategies that pharmaceutical corporations employ to safeguard their corporate interest. The strategies identified relate to scientific capture (*n* = 28), professional capture (*n* = 16), regulatory capture (*n* = 6), media capture (*n* = 3), market capture (*n* = 4), technological capture (*n* = 2), civil society capture (*n* = 4), and others (*n* = 2).

**Conclusion:**

The scientific literature using internal documents confirmed widespread corporate influence in the pharmaceutical sector. While the academic literature used internal documents related to only a handful of products, our research results, based on ghost‐management categories, demonstrate the extent of corporate influence in every interstice of pharmaceutical markets, particularly in clinical research and clinical practice. It also allows us to better refine the conceptual categories of ghost‐management to better map corporate influence and conflict of interest.

## 1 INTRODUCTION

While it is assumed that the pharmaceutical sector relies on the production of pharmaceutical knowledge through research and development, it is important to emphasize that this production of knowledge is conducted within specific socioeconomic institutions and by commercial organizations aiming at maximizing profitability. These commercial organizations can use specific strategies to influence knowledge production and clinical practices to their benefits even when it is to the detriment of public health. In 2009, the Institute of Medicine published an influential report showing the extent of commercial influence in every dimension of the healthcare ecosystem [1]. Corporate mechanisms of influence in health have been the subject of a substantial body of literature [2, 3, 4, 5, 6]. More recently, a series of papers published in the BMJ from 2019 to 2021 analyzed the growing evidence that extensive financial relationships between industry and healthcare decision‐makers distort scientific research, clinical education, and the practice of medicine [6, 7, 8, 9, 10, 11].

When it comes to public health and healthcare research, internal company documents often serve as unique and irreplaceable sources for evidence of corporate activities in pursuit of strategic goals [12, 13, 14, 15, 16, 17, 18, 19]. These documents can inform us that strategies detrimental to the public health are often used willingly and deliberately for financial gain. Given that the pharmaceutical industry has traditionally relied heavily on secrecy and that much information regarding potential risks is often treated as confidential business information, accessing and disclosing company documents, typically obtained through litigation, can be highly challenging. [20]. In the case of pharmaceuticals, the website of the Drug Industry Document Archive (DIDA), held by the University of California in San Francisco (UCSF), gathers a collection of internal company documents that allow for a better understanding of the different corporate strategies used to expand sales at the expense of patients’ health [21]. Internal company documents detail corporate tactics and strategies that are often recurrent from one case to another. In many ways, corporate scandals unveiled by internal documents should not be considered as exceptions but symptoms of normal and systematic strategies used by drug companies to manage and promote their commercial interests.

In particular, Sismondo [22, 23, 24] refers to the systematic tactics and practices that drug companies use to shape and influence medical knowledge through different paths and actors as “ghost‐management.” While economics normally consider a company's revenue to be its compensation for its serviceability in terms of producing wealth and knowledge, the notion of ghost‐management emphasizes a blind spot in economics where companies can sometimes use behind‐the‐scenes strategies, such as a selective production of ignorance [25], to influence knowledge and practices to increase profitability even when it is to the detriment of public health and welfare. Gagnon [26, 27] expands the notion of ghost‐management beyond the influence over medical knowledge to include corporate tactics and practices to influence and capture markets, regulations and technology. Building on the four categories of corporate capture developed by Miller and Harkins [28] to analyze the alcohol lobby, Gagnon categorizes the ghost‐management strategies to maximize returns into seven categories, each associated to a type of capture influencing a specific dimension of the sector: (1) scientific capture (influencing the production of scientific knowledge); (2) professional capture (influencing the practices of healthcare professionals); (3) technological capture (influencing technological pathways); (4) regulatory capture (influencing laws and regulations); (5) market capture (capacity to develop market power or restrain competition); (6) media capture (influencing media institutions); and (7) civil society capture (influencing charities, nongovernmental organizations, trade unions and other groups associated to civil society). Contrary to Carpenter and Moss [29], who favor a narrower definition of the term “capture,” the notion of capture is used here in a broader sense: capture is the aim of the ghost‐management strategies employed to gain influence or develop corporate power, and does not imply that a complete capture was necessarily achieved. Strategies of capture to influence and control a dimension of the sector in favor of commercial interests can be based on many different types of activities, such as misleading advertising, nondisclosure of relevant data, the creation of conflicts‐of‐interest to influence professional decision‐making, mergers and acquisitions, monopolization of technology, and so on.

However, although the scientific literature has used internal company documents to analyze specific corporate tactics and strategies for specific products, these documents have not been systematically analyzed to identify and categorize the different ongoing corporate strategies in the pharmaceutical sector. A systematic review of all publicly available internal company documents to identify all strategies of ghost‐management would be a colossal task beyond the scope of what can be achieved by independent researchers. This article carries out, instead, a scoping review exploring what the scientific literature learned about ghost‐management strategies in the pharmaceutical sector through internal company documents. In this regard, we consider the pharmaceutical sector to include not only research‐based pharmaceutical companies, but also generic manufacturers, pharmacy chains, distributors, and wholesalers.

This paper conducts a scoping literature review of previous scientific research that used internal pharmaceutical documents to examine and explore the ways and means drug companies used to advance their corporate interests. Through the scoping review, we want to find out how did the scientific literature use internal documents: What types of research questions those articles asked? Which methods were used? Which products were involved? Finally, what kind of patterns emerge from this research in terms of categorizing strategies used by drug companies?

In comparison to Wieland et al. [12], our objective is not only to identify all scientific articles that used internal company documents and to detail potential sources of internal data, but our paper also attempts to systematically categorize what was learned through these documents by identifying the nature of tactics and strategies at play. This scoping review can be considered as a first step in a broader research project that aims not only at detailing ongoing corporate strategies in the pharmaceutical sector, but also to compare and contrast these strategies with those occurring in other industries.

## 2 METHODS

Studies included in the scoping review had to be peer‐reviewed articles, book chapters or conference proceedings related to pharmaceutical issues in which internal company documents were explicitly referred to as the source of data or information examined in the studies. We took into account internal company documents such as correspondence, research results, targeted marketing scripts, and any other company records not initially intended for public use. We excluded papers that only examined documents prepared for external entities, such as corporate websites, advertising, gray literature, clinical literature, and corporate‐sponsored journal articles.

### 2.1 Initial scoping research methods

First, we searched eight databases, *Pubmed, Web of science, PAIS Index, Business Source, CINAHL, ERIC, Political Science Database (Pro‐Quest), and SCOPUS*, for peer‐reviewed articles, book chapters, or conference proceedings that examine or identify pharmaceutical corporations' ghost‐management strategies using internal documents. Second, building on a previous scoping review [12] with which we were able to compare our search methods and results, we selected four sets of targeted search keywords to identify relevant literature that examines leaked internal corporate documents from past litigations linked to pharmaceutical industries, companies, and specific drugs. Using four sets of search keywords, we found 6946 results from those eight databases (see Supporting Information: Appendix 1). Four sets of search keywords help capture a wide range of peer‐reviewed articles before narrowing down our scope of review. Third, snowballing on previous results, we searched the same eight databases for journal articles, book chapters or conference proceedings examining 13 drug products/drug companies for which we found internal company documents available to the public due to litigation: Aduhelm (aducanumab), Vioxx (rofecoxib), Oxycontin (oxycodone), Zyprexa (olanzapine), Seroquel (quetiapine), Paxil (paroxetine), Premarin/Prempro (conjugated estrogens), Mediator (benfluorex), Neurontin (gabapentin), and Sovaldi (sofosbuvir). We also included the more dated case of Diuril (chlorothiazide), for which documents were obtained through the release of corporate archives. To ensure a relatively complete set of literature, we also use a purposive snowball sampling method [30] to extract relevant case studies and peer‐reviewed journal articles from relevant articles’ reference lists if our search keywords fail to capture them. All results are limited to peer‐reviewed articles, book chapters, or conference proceedings researching pharmaceutical corporations' ghost‐management strategies; however, this initial scoping research still generated too many results to review in one paper, and some are not highly relevant research focuses. So, we refined our search keywords and narrowed down the scope of our review.

### 2.2 Refined scoping research methods

Based on initial research results and to avoid erratic results obtained through ERIC and Pro‐Quest, we narrowed search databases from eight to six: PubMed, Scopus, Web of Science, CINAHL, Business Source Complete, and PAIS, for peer‐reviewed articles analyzing pharmaceutical corporations' dynamic ghost‐management strategies using its internal documents. We also refined the combination of search keywords to cover broader results analyzing the pharmaceutical industry and identified articles that can only be extracted from the databases with keywords in the plural. We only selected one set of research keywords that generated the highest and most relevant results that fit our research agenda (see Supporting Information: Appendix 2). These results were generated on December 12, 2021, and different researchers on the team cross‐checked to ensure the results were accurate and reproducible. We updated the results on February 19, 2022, to ensure the results are most relevant and up to date. To capture research from diverse regions and investigate various pharmaceutical corporations, we included research from all languages and journals. Overall, among 248 articles we retrieved from six databases identified above with one set of research keywords, there are 218 peer‐reviewed journal articles in total analyzing ghost‐management mechanisms in 11 different regions in the world.

### 2.3 Initial screening

We documented all 218 peer‐reviewed journal articles published from 1973 to 2021 in an Excel pivot table template that the research team manually filled out the following information: database (one of the six databases), region (the focus of research in one or various areas), year (when peer‐reviewed articles were published), title, drug/drug companies (specific drug/drug companies or entire pharmaceutical industry), method (as how it stated in the article), results (as how it stated in the article), use internal documents (Y/N), project relevance (Y/N), decisions (whether or not we include this article: Y/N), and ghost‐management strategies (categorized based on the seven categories proposed by Gagnon [26, 27], to which we added the category “other” in case we found new types of strategies that did not fit in existing categories). Please see Supporting Information: Appendix 3 for our detailed coding mechanisms used by all researchers who coded and conducted an independent review. We also made this workbook publicly available and free of cost through Dataverse (https://borealisdata.ca/dataverse/Ghost-Management), so others can replicate our results or apply the same method to their research.

The team used two simple screening criteria to determine if the article fit the scope of our research. First, the retrieved article needs to use internal documents to examine the pharmaceutical industry/companies/products as their research method. Internal documents are defined as documents with restricted public access or no access outside of acts of whistleblowing, leaks, or court orders. We excluded documents that were initially prepared for public access, such as corporate websites. We kept this definition relatively wide to capture diverse pharmaceutical ghost‐management analyses out there using internal documents. Second, the retrieved articles needed to investigate one or many ghost‐management strategies that pharmaceutical companies incorporate to influence public policy, media coverage, and scientific results for their corporate interests. We only selected the article if it met both criteria. Two researchers reviewed the abstract and method section of the retrieved articles separately and then compared to see if screening results were consistent. After screening and removing irrelevant and duplicate journal articles across six databases, we had 52 journal articles to code and review in detail.

### 2.4 Data synthesis and analysis

After several rounds of coding, screening, and double reviewing process, we included 37 peer‐reviewed papers in the final results. All the articles included in the final results are published in English. Two researchers independently documented their review and coding decisions in the same Excel pivot table template and then discussed the results and differences in the coding process to reach a final decision. Both researchers conducted a sentence‐by‐sentence review to identify specific ghost‐management strategies at play. Data coding and extraction results are compared, discussed, and agreed upon among all researchers over Zoom meetings.

### 2.5 Patient and public involvement

No patients were involved in this research.

## 3 RESULTS

**Figure 1:**
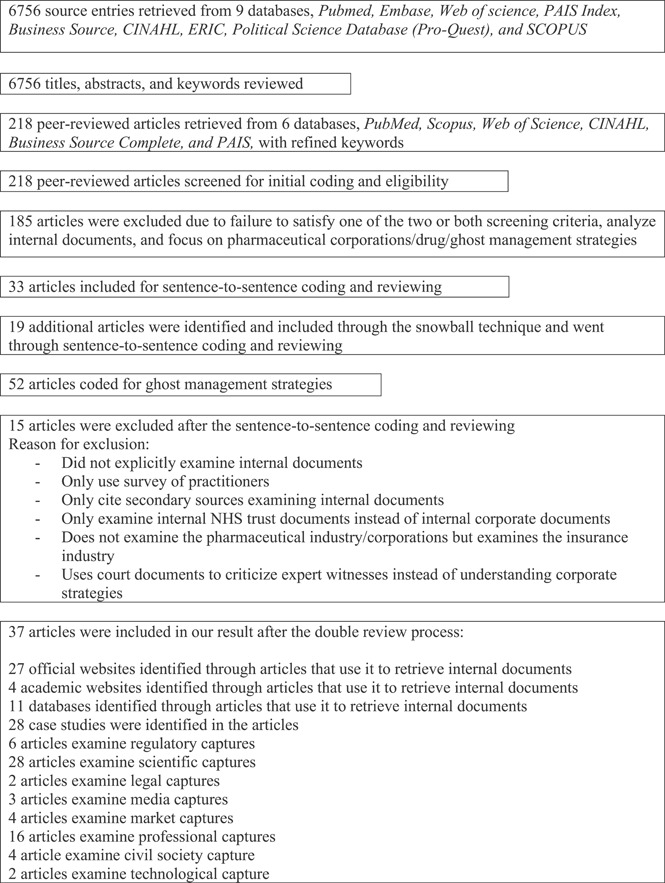
Flow chart of scoping review process.

Out of the 52 articles coded for ghost‐management strategies, we agreed to take 15 papers out of the result since they either discussed another neighboring industry (e.g., the insurance industry or the tobacco industry) or did not explicitly examine internal corporate documents but merely mentioned it (see Figure 1 for exclusion process and reasons; the full list of papers included and excluded is available in Supporting Information: Appendix 4).

Table 1 synthesizes the identification of strategies of captures examined in the 37 selected papers.

**Table 1:** Overview of strategies of captures in 37 articles.

Article	Regulatory	Scientific	Media	Market	Professional	Civil society	Technological	Other
Healy [31]		Y						
Psaty et al. [32]		Y						
Greene [33]	Y	Y			Y			
Bernschneider‐Reif et al. [34]							Y	
Breggin [35]		Y						Y
Steinman et al. [15]		Y			Y			
Devlin et al. [36]	Y	Y	Y		Y			
Gøtzsche et al. [37]		Y						
Steinman et al. [38]					Y			
Jureidini et al. [39]		Y						
Hill et al. [40]					Y	Y		Y
Psaty and Kronmal [41]		Y				Y		Y
Ross et al. [42]		Y			Y			
Matheson [43]		Y						
Jureidini and McHenry [39]		Y			Y			
Applbaum [44]	Y	Y	Y	Y	Y	Y	Y	
Landefeld and Steinman [45]		Y			Y			
Spielmans [14]					Y			
The PLoS Medicine Editors [46]		Y						
Vedula et al. [47]		Y						
Adamski et al. [48]	Y							Y
Fugh‐Berman [49]		Y						
Ross et al. [50]		Y						
Spielmans and Parry [51]		Y			Y			
Jureidini and McHenry [52]		Y						
Krumholz et al. [53]		Y		Y	Y	Y		
Matheson [54]		Y						
Coleman [55]	Y			Y	Y			Y
Vedula et al. [56]		Y						
Sismondo [57]					Y			
United States Senate Finance Committee [58]		Y						
Vedula et al. [16]		Y						
Wieland et al. [12]		Y						
Vilhelmsson et al. [59]	Y		Y	Y	Y			Y
Whitstock [60]		Y			Y			
Jayanti [61]		Y			Y			
Mulinari et al. [62]	Y	Y		Y	Y			

### 3.1 Methods used

All 37 articles use qualitative content analysis, and 11 articles use multiple research methods, including quantitative analysis [31, 38, 42, 59], literature review [12, 14, 48, 51, 53, 62], case studies using conflict of interest (COI) framework [61], comparative analysis [59], systematic/structured review [59], and standard coding methodology [42]. Articles that use literature review as a method not only analyze internal corporate documents, but also build their findings on other articles that have analyzed internal corporate documents. Identified articles used four types of qualitative and quantitative analysis software and tools, *MAXQDA* [41], IBM SPSS 25.0 [63], SAS statistical software [41, 64], and EndNote [65].

### 3.2 Sources for internal industry documents

Almost all articles obtained most of their internal document data through legal proceedings, such as litigation [14, 15, 16, 31, 32, 34, 35, 36, 37, 38, 39, 41, 42, 43, 44, 46, 47, 48, 49, 50, 51, 52, 53, 54, 55, 56, 57, 59, 60, 61, 62, 66], Drug Industry Document Archive (DIDA) [12, 45, 49], or Public Access to Court Electronic Records (PACER) [12, 55]. One article only used documents made accessible through corporate archives [33]. Another article is a revised version of a US Senate Finance Committee Report analyzing 5000 documents made available at the request of the Chair of the Committee [58]. Twenty‐two articles used more than one type of resource and document for their analysis. Seven articles used internal and external corporate documents [45, 46, 53, 61], such as Internal marketing documents [36], corporate statements [55], industry journals [33], and gray literature to identify ongoing corporate strategies [31, 48, 57]. Four articles found their data through whistleblowers [35, 52, 59, 62], and 16 articles supplemented their data analysis through published clinical trial data [16, 41, 47, 56], interviews [57], personal communication [57], literature review of previous research [12, 41, 48, 51, 54, 61], the FDA's online drug approval package (DAP) database [41, 60], DEA press statement [55], SEC filings [55], and corporate archives that are readily accessible to researchers who requested to view them [32, 33, 34, 38, 42] (see Supporting Information: Appendix 5).

### 3.3 Ghost‐management strategies

All 37 articles unveil dynamic ghost‐management strategies that pharmaceutical corporations employ to safeguard their corporate interest. The category of scientific capture is by far the most analyzed in the scientific literature. Twenty‐eight articles unveil the dynamics of scientific capture, which we coded into five subcategories. First, 19 articles reveal strategies of ghostwriting/publication planning issues that pharmaceutical corporations employ [16, 31, 33, 37, 39, 42, 43, 44, 45, 47, 49, 51, 52, 54, 56, 58, 60, 61, 66]. Second, 16 articles specifically mention the nondisclosure/selective reporting issues [12, 15, 16, 32, 39, 41, 42, 46, 47, 50, 51, 52, 53, 56, 60, 66]. Third, nine articles identify how pharmaceutical corporations downplay negative results in clinical trials [16, 39, 47, 51, 52, 53, 56, 58, 66]. Fourth, six articles have studied bias in clinical trial design [15, 44, 53, 56, 60, 66]. Last but not least, seven articles mention the dynamics of COI in research [36, 37, 42, 52, 54, 58, 66], including one article that looks explicitly into the COI issue of journals/publishers [52]. Following the definition provided the Institute of Medicine [1], a COI can be understood as “a set of circumstances that creates a risk that professional judgment or actions regarding a primary interest will be unduly influenced by a secondary interest.” COI are included in categories of capture when the set of circumstances is created by the actions of industry. Half of the articles reveal more than one type of scientific capture in their article.

Professional capture is another commonly examined ghost‐management strategy among our selected articles. 16 articles identify dynamics of professional capture, which we coded into nine subcategories of capture: key opinion leaders (KOL) [15, 36, 44, 45, 57, 61, 62, 66], detailing/promotional meetings [14, 33, 36, 38, 44, 53, 55, 62], off‐label promotion [14, 38, 44, 45, 59, 62, 66], seeding trials [42, 53, 60, 61], clinical education/training [15, 45, 61], advertising to health care professionals [33], COI in clinical practice guidelines [61], gifts and bribes [44, 53]. Eleven articles have identified more than one professional capture, and one article analyzed six types of professional captures in one article [44].

Six articles identified four kinds of regulatory captures: lobbying efforts [33, 36, 44], self‐regulation [55, 59, 62], COI of regulators [48, 62], and revolving doors [33]. Three articles mention strategies of media capture: direct‐to‐consumer advertising (DTCA) [44, 59] and direct collaboration with journalists [36]. Another four articles document strategies of market capture: market concentration [44, 53, 55], chargeback [55, 62], influence over reimbursement decisions [59], and unlawful commerce [55]. Another two articles investigate the strategic patenting issue in technological capture [34, 44]. Four articles identify the type of civil society capture based on COI with patient groups [41, 44, 53], misinforming patients during recruitment [40, 41], and astroturfing [44]. Finally, we identified ghost‐management strategies that cannot be included in existing categories and were thus categorized as “other”: willful unlawful activity [55] and preventing whistleblowing through intimidation [35].

## 4 DISCUSSION

Internal pharmaceutical industry documents include relevant information and qualitative analysis on promotional activities, ghostwriting practices, bias in clinical trial design or COI in different dimensions of the health care delivery related to pharmaceuticals. It also contains quantitative and other data related to clinical trials, allowing the identification of selective reporting or non‐disclosure of adverse effects, in particular in the cases of Baycol (cerivastatin) [32], enzalutamide (Xtandi) [62], Vioxx (rofecoxib) [41, 42, 50, 60], Zyprexa (olanzapine) [51], Neurontin (gabapentin) [15, 16, 47, 53, 56] or Paxil (paroxetine) [35, 39, 66]. The use of the ghost‐management framework and the taxonomy in terms of seven different types of capture allows a better understanding the overall impact of the sum of these activities in each dimension of the sector.

Internal pharmaceutical industry documents were mostly obtained through court litigations. Many of these documents allow exploring the ethically problematic relationships between industry and different stakeholders such as health care professionals, medical faculties and clinical training, clinical practice guidelines, medical journals, and distributors. Of particular interest, we find a handful of products for which internal documents were made available and allowed exploring the extent of ghost‐management practices: Diuril (chlorothiazide), Baycol (cerivastatin), Vioxx (rofecoxib), Zyprexa (olanzapine), Paxil (paroxetine), Premarin/Prempro (conjugated estrogens), Neurontin (gabapentin), and enzalutamide (Xtandi).

A simple case study such as Zyprexa (olanzapine) produced by Eli Lilly to treat schizophrenia and “complicated moods” shows how heavy corporate influence is found in every dimension of clinical research and practice when creating a pharmaceutical blockbuster. The scientific narratives for the product were managed from the start by publication planning companies [44, 51, 61], ghostwriting scientific articles in medical journals to provide strategic narratives for the company's sales force. Eli Lilly promoted to physicians new conditions that could be treated with Zyprexa (olanzapine), and it also used selective reporting of its trial data and downplayed negative results [51]. Detailed documents about the marketing mix for the product showed the extensive use of off‐label promotion, key opinion leaders, detailing and promotional meetings [14, 51] in which tailored messages, scripted patient profiles, and psychological profiling of physicians were used by sales representatives [14, 44, 51]. In addition to the marketing mix, the company used seeding trials and was heavy‐handed on the influence of clinical education and training about the disease and the product and on the drafting of clinical practice guidelines [61].

Another case study on the promotion of Diuril (chlorothiazide) against hypertension by the company Merck in the 1950s and 1960s shows that similar strategies of influence on the medical profession already existed at that time [33]. The case of Diuril (chlorothiazide) shows that the company used ghostwriting tactics, massive advertising to healthcare professionals and the use of detailing and promotional meetings. It also used significant lobbying efforts and revolving doors to transform the way the medical profession considers and treats hypertension: “marketing is not merely an endpoint, but a phenomenon that suffuses the entire process; drug defines disease at least as much as disease defines and elicits drug”; and the result for Diuril (chlorothiazide) was successful considering that “Diuril lowered the thresholds for the prescription and consumption of antihypertensive medications, enlarged the population of potential hypertensive patients in both clinical trials and clinical practice, and contributed to the consolidation of a single, mobile threshold for the definition of hypertension” [33].

The low number of specific cases analyzed through internal documents should not be interpreted as meaning that these corporate strategies are exceptional and do not represent the norm of ongoing dynamics for pharmaceutical products. The low number of cases should be interpreted, instead, in terms of the difficulty of accessing such documents. In the past, testimonies of sales representatives’ managers [67], sales representatives [68], professional ghostwriters [43], pharmaceutical executives [69], as well as in‐depth analyses of the workings of knowledge production for pharmaceuticals [22, 70, 71] have shown how widespread ghost‐management strategies can be for different types of products. In terms of criminal and civil penalties, on average, there were 34 settlements every year in the United States between 2008 and 2017 [72]. In fact, this handful of case studies unveils the existence of a series of stakeholders and actors for which these problematic behind‐the‐scenes strategies of corporate influence are the normal way of doing things.

The comparative analyses in the identified papers reveal that strategies observed in the marketing and publication of specific pharmaceutical products are not isolated incidents but are instead widespread across the industry. Among the 37 papers we identified, some of the papers made parallels with strategies observed with other pharmaceutical products. For example, a paper on ghostwriting and publication planning in the case of hormone replacement therapy explains that the same dynamics were found for drugs such as Paxil (paroxetine), Pondimin (fenfluramine) and Lonamin (phentermine), Neurontin (gabapentin), Vioxx (rofecoxib), and Zoloft (sertraline). The analysis of the marketing of Zyprexa (olanzapine) shows that the same strategies are applied to other drugs such as Seroquel (quetiapine), Cymbalta (duloxetine), Xigris (drotrecogin alfa), Xyrem (sodium oxybate), or blockbuster antidepressants [44, 51, 61]. The analysis of the design of seeding trials for Vioxx (rofecoxib) showed that the same strategies were applied for Arcoxia (etoricoxib) [60]. When given access to internal documents for the bone growth product Infuse, the US Senate Finance Committee discovered “troubling evidence that Medtronic officials influenced the content of articles in peer‐reviewed scientific publications to present InFuse in the best possible light” using strategies of ghostwriting and publication planning, as well as downplaying negative results [58].

COI, as defined in terms of sets of circumstances created by the actions of the industry and creating a risk that professional judgment or actions regarding a primary interest will be unduly influenced by a secondary interest, seem to be a central element in different categories of capture. The scientific literature, using internal documents, confirmed the widespread nature of COI in the pharmaceutical sector, while also offering additional insights about the mechanisms through which these COI become detrimental to public health. COI was often identified in clinical research [37, 40, 43, 52, 58, 66], in clinical education and training [15, 45, 61], and in clinical practice guidelines [61]. COI was also found in the interaction of the industry with regulators [48, 62], payers [59], journalists [36], patient groups [41, 44, 53], and distributors/wholesalers [55]. Some of the papers described how some actors and stakeholders existed as a results of ghost‐management strategies, such as conflicted key opinion leaders [15, 36, 40, 45, 51, 57, 61, 66] and medical writing agencies [46, 51], such as DesignWrite, Parthenon, PeerView, Sunvalley Communication, Dianthus Medical, and Current Medical Directions.

An interesting result was that two articles did not fit into any of the previously identified ghost‐management categories and were thus classified as “other.” In other category, one article detailed willful and unlawful activity [55], while the other documented the prevention of whistleblowing through intimidation [35]. Because these two strategies relate to legal issues, it could be of interest to explore the possibility of creating a new category for ghost‐management strategies specific to legal issues.

The analysis of internal company documents offers unique perspectives on behind‐the‐scenes efforts and strategies used by drug companies to shape and influence knowledge, narratives, and prescribing habits to favor their commercial interests. The analysis of these 37 documents of the scientific literature allows a much better understanding of how corporate influence is widespread in healthcare practices, but this literature remains limited. A 2014 study [12] identified 20 scientific papers that used internal pharmaceutical industry documents, while it also identified 325 scientific papers that used internal tobacco industry documents. An important limitation in our analysis is the restraining of results to peer‐reviewed documents, while leaks and internal documents are more often disclosed by investigative journalism and lawyers during litigation. Also, medical and scientific articles tend to focus mostly on the form of capture (scientific and professional) that relates directly to health research and the medical profession. We also did not include trial restoration studies based on the disclosure of clinical trial information in our research scope, but this type of research could potentially yield fruitful discoveries. Furthermore, there are more court cases and studies examining those released internal corporate documents after our research timeline, especially after the massive disclosure in 2022 of internal industry documents related to opioid drugs [73, 74]. Another limitation is that we did not analyze internal documents directly. We simply synthesized what was said in the scientific literature.

By narrowing our research only to the scientific literature, we nevertheless end up with a solid foothold, even if limited, to understand the ways and means of corporate influence over clinical research and practice, as well as in the organization of pharmaceutical markets. Analyzing the content of the scientific literature using internal company documents based on categories of ghost‐management allows one to better understand how pervasive and “normal” corporate influence on pharmaceutical knowledge and practice has become. A more systematic disclosure of internal company documents could confirm the extent of these actual practices and could transform the way academics and researchers analyze the industry. Public authorities can also directly investigate different corporate practices and request internal company documents when necessary, like in the case of INFUSE trials [58]. In particular, it will be interesting to see how the recent massive disclosure of internal documents through the Opioids Internal Documents Archive (OIDA) might allow a more detailed analysis of on‐going corporate practices and provide potential regulatory solutions [75]. Future research should analyze internal documents directly and include journalistic investigations, criminal investigations, trial restoration studies or regulatory examinations to provide more depth in the understanding of corporate influence. By detailing even more ghost‐management categories, future research could allow developing an analytical framework to understand what has become business as usual in the pharmaceutical sector.

## AUTHOR CONTRIBUTIONS

Marc‐André Gagnon and Miaoran Dong both substantially contributed to the conceptualization, the design and the methods of the scoping review; to the acquisition, analysis, and interpretation of data for the work. They drafted the scoping review together (original draft as well as revisions) and gave final approval for its publication. They are both accountable for all aspects of the work in ensuring that questions related to the accuracy or integrity of any part of the work are appropriately investigated and resolved. Marc‐André Gagnon was responsible for the funding acquisition for the project, as well as its supervision and administration. Marc‐André Gagnon and Miaoran Dong would like to acknowledge the help of Grace Dupasquier, who acted as a research assistant for this paper and provided technical support with the analysis of the texts and the editing of the article.

## CONFLICTS OF INTEREST STATEMENT

We have no known conflict of interest to disclose except that MAG acted as an expert witness for Justice Canada on a case relating to patented drug prices. This research is funded by the Social Sciences and Humanities Research Council (SSHRC).

## ETHICS STATEMENT

Ethics approval was not required since the research did not involve human participants or animal research.

## Supporting information

Supporting information.

## Data Availability

The data that support the findings of this study are openly available in *Dataverse* at https://borealisdata.ca/dataverse/Ghost-Management. This article has earned Open Data and Open Materials badges. Data and materials are available at https://borealisdata.ca/dataset.xhtml?persistentId=doi:10.5683/SP3/MR77HT.
