# Supplementary material for: What did the scientific literature learn from internal company documents in the pharmaceutical industry? A scoping review
Source: Cochrane Evid Synth Methods. 2023 Apr 27;1(3):e12011. doi: 10.1002/cesm.12011 (PMC11795944; doi:10.1002/cesm.12011)
Supplement: Supplementary file 1 — Supporting information. [file CESM-1-e12011-s001.docx]

**Appendix 1**

**Initial search results for identifying peer-reviewed journal articles using internal company documents for pharmaceutical research in nine databases**

| **Databases** | **Drug industry_wide search**  ("pharmaceutical” OR “pharmaceuticals" OR "drug industry" OR "pharmaceutical Industry" OR “drug industries” OR “pharmaceutical Industries”)  AND ("Confidential documents" OR "public policy" OR "leaks" OR "whistleblowing" OR "whistleblow" OR "criminal pleas" OR "criminal investigations" OR "Internal document" OR "court documents" OR "pleadings" OR "expert testimony") | **Drug industry_internal documents**  ("pharmaceutical” OR “pharmaceuticals" OR "drug industry" OR "pharmaceutical Industry" OR “drug industries” OR “pharmaceutical Industries”)  AND (« Internal document » OR « Confidential document » OR « court document » OR « corporate document ») | **Drug_Wide**  (« Diuril » OR « Aduhelm » OR « Vioxx » OR « Oxycontin » OR « opioid by Purdue » OR « Zyprexa » OR « Seroquel » OR « Paxil » OR « Premarin » OR « Mediator » OR « Neurontin » OR « gabapentin Hormone replacement therapy » OR « Prempro » OR « Sovaldi » OR « Seroquel » OR « Paxil ») AND (« Confidential documents » OR « public policy » OR « leaks » OR « whistleblowing » OR « whistleblow » OR « criminal pleas » OR « criminal investigations » OR « Internal document » OR « court documents » OR « pleadings » OR « expert testimony ») | **Drug_Internal Document**  ("Diuril" OR "Aduhelm" OR "Vioxx" OR "Oxycontin" OR "opioid by Purdue" OR "Zyprexa" OR "Seroquel" OR "Paxil" OR "Premarin" OR "Mediator" OR "Neurontin" OR "gabapentin Hormone replacement therapy" OR "Prempro" OR "Sovaldi" OR "Seroquel" OR "Paxil") AND ("Internal document" OR "Confidential document" OR "court document" OR "corporate document") |
| --- | --- | --- | --- | --- |
| Business source | 2,155  (Trade Publications, 684; **Academic Journals, 633**; Magazines, 498; Newspapers, 211; Industry Profiles, 41; Market Research Reports, 11Product Reviews, 3; Country Reports, 2) | 4  (Newspapers, 2; **Academic Journals, 1**; Magazines, 1) | 114  (**Academic Journals, 66**; Magazines, 15; Trade Publications, 12; Newspapers, 12; Market Research Reports, 1) | 1  (Magazines, 1) |
| CINAHL | 161  (**Academic Journals, 148**; Magazines, 9; CEUs, 3) | 0 | 21  (**Academic Journals, 20**; Magazines, 1) | 0 |
| ERIC | 7  (Scholarly Journals, 6;Government & Official PublicationsGovernment & Official Publications, ‎1) | 0 | 18  (Scholarly Journals‎, 13; Reports‎, 3; Speeches & Presentations‎, 2) | 0 |
| PAIS | 400  (**Scholarly Journals‎, 348**; Books‎, 47; Magazines‎, 2; Reports‎, 2;Trade Journals‎, 1) | 1  **Academic Journals, 1** | 23  **Academic Journals, 23** | 0 |
| Political science database | 435  (**Scholarly Journals,‎ 221**; Other Sources‎, 80; Blogs, Podcasts, & Websites‎, 54; Magazines‎, 38; Wire Feeds‎, 31;Reports‎, 8;Newspapers‎, 2;Trade Journals‎, 1) | 0 | 24  (**Scholarly Journals‎, 16**; Blogs, Podcasts, & Websites‎, 5; Dissertations & Theses‎, 2; Other Sources‎, 1) | 0 |
| **Pubmed** | **1,327** | **166** | **43** | **13** |
| SCOPUS | **2,271**  (**Article, 1,435**; Review, 382; Note, 121; Conference Paper, 81; Editorial, 68; Book Chapter, 55; Short Survey, 49; Letter, 43; Book, 21; Erratum,11;Conference Review,5) | 29  (Article, 19) | 233  (**Article, 170**; Review, 26; Conference Paper, 17; Book Chapter, 7; Note, 4; Book, 3; Short Survey, 3; Editorial2; Undefined, 1) | 11  (**Article, 5)** |
| Web of science | 2828 | 55 | 0 | 0 |

**Appendix 2**

**Search keywords to identify peer-reviewed journal articles using internal company documents for pharmaceutical research**

("pharmaceutical” OR “pharmaceuticals" OR "drug industry" OR "pharmaceutical Industry" OR “drug industries” OR “pharmaceutical Industries”)

AND

("internal document" OR "confidential document" OR "court document" OR "corporate document" OR "internal documents" OR "confidential documents" OR "court documents" OR "corporate documents" OR "leaked document" OR "leaked documents")

**Appendix 3**

**Coding mechanisms for research focus:**

**Analysing/identifying pharmaceutical corporations’ ghost management strategies**: (1) **Regulatory** [Conflicts of interests (COI) of regulators, self-regulation, lobbying efforts, revolving doors]; (2) **Scientific** [COI in research (COI of investigators, COI of authors, COI of journals/publishers), non-disclosure/selective reporting, ghostwriting/publication planning (disease mongering), bias in clinical trial design, downplaying negative results; (3) **Media** [collaboration with journalists (COI in social network/“social production of information”), direct-to-consumer advertising (DTCA)]; (4) **Market** [influencing reimbursement decisions (COI of payers), unlawful commerce, market concentration, chargeback]; (5) **Professional** [detailing/promotional meetings (psychological profiling, scripted patient profiles, tailoring of message, relationships), Key Opinion Leaders (KOL), seeding trials, off-label promotion, medical education/training (continuing medical education, educational grants), COI in Clinical Practice guidelines, COI of health care professionals, advertising to health care professionals, gift and bribe]; (6) **Civil Society** [misinforming patients in recruitment, COI with patient groups (astroturfing)]; (7) **Technological** [strategic patenting]; (8) **Other captures**

**Coding mechanisms for analysis:**

**Data Analysis**: (1) qualitative [qualitative content analysis]; (2) quantitative analysis; (3) other (please specify)

**Coding mechanisms for internal documents:**

**Types of data collection**: (1) legal proceedings (leaked documents, litigation, etc.); (2) Corporate public documents (financial documents, advertisements, marketing material, etc.); (3) Whistleblower; (4) Other (please specify)

**Sources of data collection**: please specify

**Coding mechanisms for results:**

Open-ended

**Appendix 4**

**51 peer-reviewed articles used in sentence-to-sentence review (37 included, 15 excluded)**

**Appendix 4.1**

**37 peer-reviewed articles included for the final results:**

1. Vilhelmsson A, Davis C, Mulinari S. Pharmaceutical Industry Off-label Promotion and Self-regulation: A Document Analysis of Off-label Promotion Rulings by the United Kingdom Prescription Medicines Code of Practice Authority 2003–2012. PLoS Med [Internet]. 2016 Jan 26 [cited 2021 Nov 29];13(1):e1001945. Available from: <https://dx.plos.org/10.1371/journal.pmed.1001945>

2. Psaty BM, Furberg CD, Ray WA, Weiss NS. Potential for conflict of Interest in the evaluation of suspected adverse drug reactions use of cerivastatin and risk of rhabdomyolysis. JAMA [Internet]. 2004 Dec 1 [cited 2021 Oct 25];292(21):2622–31. Available from: https://doi.org/10.1001/jama.292.21.2622

3. Landefeld CS, Steinman MA. The neurontin legacy — marketing through misinformation and manipulation. New England Journal of Medicine [Internet]. 2009 Jan 8 [cited 2022 Jan 31];360(2):103–6. Available from: https://doi.org/10.1056/NEJMp0808659

4. Fugh-Berman AJ. The haunting of medical journals: How ghostwriting sold “HRT.” PLoS Med [Internet]. 2010 Sep 7 [cited 2022 Feb 6];7(9):e1000335. Available from: https://www.ncbi.nlm.nih.gov/pmc/articles/PMC2935455/

5. Psaty BM, Kronmal RA. Reporting mortality findings in trials of rofecoxib for alzheimer disease or cognitive impairment: A case study based on documents from rofecoxib litigation. JAMA [Internet]. 2008 Apr 16 [cited 2021 Oct 25];299(15):1813–7. Available from: https://doi.org/10.1001/jama.299.15.1813

6. Ross JS, Madigan D, Konstam MA, Egilman DS, Krumholz HM. Persistence of cardiovascular risk after rofecoxib discontinuation. Archives of Internal Medicine [Internet]. 2010 Dec 13 [cited 2022 Feb 6];170(22):2035–6. Available from: https://doi.org/10.1001/archinternmed.2010.461

7. Bernschneider-Reif S, Úxler F, Freudenmann RW. The origin of MDMA (“Ecstasy”) – separating the facts from the myth. 2006;7.

8. Matheson A. Corporate Science and the Husbandry of Scientific and Medical Knowledge by the Pharmaceutical Industry. BioSocieties [Internet]. 2008 Dec [cited 2022 Feb 7];3(4):355–82. Available from: http://www.cambridge.org/core/journals/biosocieties/article/corporate-science-and-the-husbandry-of-scientific-and-medical-knowledge-by-the-pharmaceutical-industry/92DB12151B1EF9CE722491074F36CF7E

9. Healy D, Cattell D. Interface between authorship, industry and science in the domain of therapeutics. The British Journal of Psychiatry [Internet]. 2003 Jul [cited 2022 Feb 6];183(1):22–7. Available from: http://www.cambridge.org/core/journals/the-british-journal-of-psychiatry/article/interface-between-authorship-industry-and-science-in-the-domain-of-therapeutics/C6C9C63588A474F6CF6CA27BA2BD43A6

10. Steinman MA, Harper GM, Chren M-M, Landefeld CS, Bero LA. Characteristics and impact of drug detailing for gabapentin. PLoS Med. 2007 Apr;4(4):e134.

11. Breggin PR. Court filing makes public my previously suppressed analysis of Paxil’s effects. Ethical Hum Psychol Psychiatry. 2006;8(1):77–84.

12. Applbaum K. Getting to yes: corporate power and the creation of a psychopharmaceutical blockbuster. Cult Med Psychiatry. 2009 Jun;33(2):185–215.

13. Matheson A. How Industry Uses the ICMJE Guidelines to Manipulate Authorship—And How They Should Be Revised. PLOS Medicine [Internet]. 2011 Aug 9 [cited 2022 Jan 31];8(8):e1001072. Available from: https://journals.plos.org/plosmedicine/article?id=10.1371/journal.pmed.1001072

14. Gøtzsche PC, Hróbjartsson A, Johansen HK, Haahr MT, Altman DG, Chan A-W. Ghost Authorship in Industry-Initiated Randomised Trials. PLOS Medicine [Internet]. 2007 Jan 16 [cited 2022 Jan 31];4(1):e19. Available from: https://journals.plos.org/plosmedicine/article?id=10.1371/journal.pmed.0040019

15. Steinman M, Bero LA, Chren M, Landefeld CS, Steinman MA, Bero LA, et al. Narrative review: the promotion of gabapentin: an analysis of internal industry documents. ANN INTERN MED [Internet]. 2006 Aug 15 [cited 2021 Dec 10];145(4):284–93. Available from: https://proxy.library.carleton.ca/login?url=https://search.ebscohost.com/login.aspx?direct=true&db=cin20&AN=106206471&site=ehost-live

16. Coleman JJ. The Supply Chain of Medicinal Controlled Substances: Addressing the Achilles Heel of Drug Diversion. Journal of Pain & Palliative Care Pharmacotherapy [Internet]. 2012 Sep 4 [cited 2021 Dec 13];26(3):233–50. Available from: http://www.tandfonline.com/doi/full/10.3109/15360288.2012.703294

17. Spielmans GI. The promotion of olanzapine in primary care: an examination of internal industry documents. Soc Sci Med. 2009 Jul;69(1):14–20.

18. Vedula SS, Bero L, Scherer RW, Dickersin K. Outcome Reporting in Industry-Sponsored Trials of Gabapentin for Off-Label Use. New England Journal of Medicine [Internet]. 2009 Nov 12 [cited 2021 Dec 10];361(20):1963–71. Available from: https://doi.org/10.1056/NEJMsa0906126

19. Wieland LS, Rutkow L, Vedula SS, Kaufmann CN, Rosman LM, Twose C, et al. Who Has Used Internal Company Documents for Biomedical and Public Health Research and Where Did They Find Them? PLOS ONE [Internet]. 2014 May 6 [cited 2021 Dec 10];9(5):e94709. Available from: https://journals.plos.org/plosone/article?id=10.1371/journal.pone.0094709

20. Whitstock M. Manufacturing the truth: from designing clinical trials to publishing trial data. IJME [Internet]. 2017 Nov 14 [cited 2021 Dec 10]; Available from: http://ijme.in/articles/manufacturing-the-truth-from-designing-clinical-trials-to-publishing-trial-data/?galley=html

21. Greene JA. Releasing the Flood Waters: Diuril and the Reshaping of Hypertension. Bulletin of the History of Medicine [Internet]. 2005 [cited 2021 Dec 10];79(4):749–94. Available from: https://muse.jhu.edu/article/190764

22. Hill KP, Ross JS, Egilman DS, Krumholz HM, Hill KP, Ross JS, et al. The ADVANTAGE seeding trial: a review of internal documents. ANN INTERN MED [Internet]. 2008 Aug 19 [cited 2021 Dec 10];149(4):251–8. Available from: https://proxy.library.carleton.ca/login?url=https://search.ebscohost.com/login.aspx?direct=true&db=cin20&AN=105809375&site=ehost-live

23. Adamski J, Godman B, Ofierska-Sujkowska G, Osinska B, Herholz H, Wendykowska K, et al. Risk sharing arrangements for pharmaceuticals: potential considerations and recommendations for European payers. BMC HEALTH SERV RES [Internet]. 2010 Jan [cited 2021 Dec 10];10:153–153. Available from: https://proxy.library.carleton.ca/login?url=https://search.ebscohost.com/login.aspx?direct=true&db=cin20&AN=105063687&site=ehost-live

24. Spielmans GI, Parry PI. From evidence-based medicine to marketing-based medicine: evidence from internal industry documents. J BIOETHICAL INQUIRY [Internet]. 2010 Mar [cited 2021 Dec 10];7(1):13–29. Available from: https://proxy.library.carleton.ca/login?url=https://search.ebscohost.com/login.aspx?direct=true&db=cin20&AN=105177473&site=ehost-live

25. Jayanti RK. Missing the Forest for the Trees: Marketing Systems Approach to Pharmaceutical Industry Marketing Practices. Journal of Macromarketing [Internet]. 2021 Oct 19 [cited 2021 Dec 9];1. Available from: https://proxy.library.carleton.ca/login?url=https://search.ebscohost.com/login.aspx?direct=true&db=bth&AN=153086578&site=ehost-live

26. Mulinari S, Davis C, Ozieranski P. Failure of Responsive Regulation? Pharmaceutical Marketing, Corporate Impression Management and Off-Label Promotion of Enzalutamide in Europe. J White Collar Corp Crime. 2021 Jun 1;2(2):69–80.

27. United States Senate Finance Committee. Staff report on Medtronic’s influence on INFUSE clinical studies. International Journal of Occupational and Environmental Health [Internet]. 2013 [cited 2021 Dec 3];19(2):67–76. Available from: <http://www.tandfonline.com/doi/full/10.1179/2049396713Y.0000000020>

28. Vedula SS, Li T, Dickersin K. Differences in Reporting of Analyses in Internal Company Documents Versus Published Trial Reports: Comparisons in Industry-Sponsored Trials in Off-Label Uses of Gabapentin. Kesselheim AS, editor. PLoS Med [Internet]. 2013 Jan 29 [cited 2021 Dec 3];10(1):e1001378. Available from: https://dx.plos.org/10.1371/journal.pmed.1001378

29. Sismondo S. Key Opinion Leaders and the Corruption of Medical Knowledge: What the Sunshine Act Will and Won’t Cast Light on. J Law Med Ethics [Internet]. 2013 [cited 2021 Dec 3];41(3):635–43. Available from: https://www.cambridge.org/core/product/identifier/S1073110500025122/type/journal_article

30. Ross JS, Hill KP, Egilman DS, Krumholz HM. Guest Authorship and Ghostwriting in Publications Related to Rofecoxib: A Case Study of Industry Documents From Rofecoxib Litigation. JAMA [Internet]. 2008 Apr 16 [cited 2021 Dec 3];299(15):1800. Available from: http://jama.jamanetwork.com/article.aspx?doi=10.1001/jama.299.15.1800

31. The PLoS Medicine Editors. Ghostwriting: The Dirty Little Secret of Medical Publishing That Just Got Bigger. PLoS Med [Internet]. 2009 Sep 8 [cited 2021 Nov 29];6(9):e1000156. Available from: https://dx.plos.org/10.1371/journal.pmed.1000156

32. Vedula SS, Goldman PS, Rona IJ, Greene TM, Dickersin K. Implementation of a publication strategy in the context of reporting biases. A case study based on new documents from Neurontin litigation. Trials. 2012 Aug 13;13:136.

33. Devlin E, Hastings G, Smith A, McDermott L, Noble G. Pharmaceutical marketing: a question of regulation. Journal of Public Affairs (14723891) [Internet]. 2007 May [cited 2021 Nov 15];7(2):135–47. Available from: https://proxy.library.carleton.ca/login?url=https://search.ebscohost.com/login.aspx?direct=true&db=bth&AN=25133447&site=ehost-live

34. Jureidini JN, McHenry LB. Conflicted medical journals and the failure of trust. Account Res. 2011 Jan;18(1):45–54.

35. McHenry LB, Jureidini JN. Industry-sponsored ghostwriting in clinical trial reporting: a case study. Account Res. 2008 Sep;15(3):152–67.

36. Jureidini JN, McHenry LB, Mansfield PR. Clinical trials and drug promotion: Selective reporting of study 329. International Journal of Risk & Safety in Medicine [Internet]. 2008 Jan 1 [cited 2021 Oct 25];20(1–2):73–81. Available from: <https://content.iospress.com/articles/international-journal-of-risk-and-safety-in-medicine/jrs426>

37. Krumholz SD, Egilman DS, Ross JS. Steps: A narrative account of a gabapentin seeding trial. Arch Intern Med [Internet]. 2011 Jun 27 [cited 2022 Apr 30];171(12):1100–7. https://doi.org/10.1001/archinternmed.2011.241 PMID: 21709111

**Appendix 4.2**

**15 peer-reviewed articles excluded during the sentence-to-sentence coding process:**

1. Whitehead P. Causality and collateral estoppel: Process and content of recent SSRI litigation. Journal of the American Academy of Psychiatry and the Law. 2003;31(3):377–82.
2. Henry DA, Hill SR, Doran E, Newby DA, Henderson KM, Maguire J, et al. Medical specialists and pharmaceutical industry-sponsored research: a survey of the Australian experience. Medical Journal of Australia [Internet]. 2005 [cited 2021 Dec 10];182(11):557–60. Available from: https://onlinelibrary.wiley.com/doi/abs/10.5694/j.1326-5377.2005.tb06813.x
3. Delaware Journal of Corporate Law. Unreported Cases. Delaware Journal of Corporate Law [Internet]. 2007 Sep [cited 2021 Dec 9];32(3):893–1033. Available from: https://proxy.library.carleton.ca/login?url=https://search.ebscohost.com/login.aspx?direct=true&db=bth&AN=28607402&site=ehost-live
4. Hill KP, Ross JS, Egilman DS, Krumholz HM, Hill KP, Ross JS, et al. The ADVANTAGE seeding trial: a review of internal documents. ANN INTERN MED [Internet]. 2008 Aug 19 [cited 2021 Dec 10];149(4):251–8. Available from: https://proxy.library.carleton.ca/login?url=https://search.ebscohost.com/login.aspx?direct=true&db=cin20&AN=105809375&site=ehost-live
5. Rabin A, Edelman JM, Sox HC, Rennie D, Edelman JM. ADVANTAGE: Merck does say “no”... ANN INTERN MED [Internet]. 2008 Nov 18 [cited 2021 Dec 10];149(10):774–5. Available from: https://proxy.library.carleton.ca/login?url=https://search.ebscohost.com/login.aspx?direct=true&db=cin20&AN=105582425&site=ehost-live
6. Read J. Schizophrenia, drug companies and the internet. Social Science & Medicine [Internet]. 2008 [cited 2021 Nov 29];66(1):99–109. Available from: https://linkinghub.elsevier.com/retrieve/pii/S0277953607004315
7. Woods S, Tek C, Srihari V. Adverse drug effects not detected at licensing review: Regulatory autopsy of olanzapine. In Schizophrenia Bulletin; 2011. p. 37: 30.
8. Norris SL, Holmer HK, Ogden LA, Burda BU, Fu R. Characteristics of physicians receiving large payments from pharmaceutical companies and the accuracy of their disclosures in publications: an observational study. BMC Med Ethics. 2012 Sep 26;13:24.
9. Checketts JX, Sims MT, Vassar M. Evaluating Industry Payments Among Dermatology Clinical Practice Guidelines Authors. JAMA Dermatol [Internet]. 2017 Dec 1 [cited 2021 Dec 3];153(12):1229. Available from: <http://archderm.jamanetwork.com/article.aspx?doi=10.1001/jamadermatol.2017.3109>
10. Charbonneau M, Gagnon M-A. Surviving niche busters: Main strategies employed by Canadian private insurers facing the arrival of high cost specialty drugs. Health Policy [Internet]. 2018 [cited 2021 Dec 13];122(12):1295–301. Available from: https://linkinghub.elsevier.com/retrieve/pii/S0168851018303877
11. Bastani P, Samadbeik M, Dinarvand R, Kashefian-Naeeini S, Vatankhah S. Qualitative analysis of national documents on health care services and pharmaceuticals` purchasing challenges: evidence from Iran. BMC Health Serv Res [Internet]. 2018 [cited 2021 Dec 3];18(1):410. Available from: https://bmchealthservres.biomedcentral.com/articles/10.1186/s12913-018-3261-0
12. Feldman HR, DeVito NJ, Mendel J, Carroll DE, Goldacre B. A cross-sectional study of all clinicians’ conflict of interest disclosures to NHS hospital employers in England 2015-2016. BMJ Open [Internet]. 2018 [cited 2021 Dec 3];8(3):e019952. Available from: https://bmjopen.bmj.com/lookup/doi/10.1136/bmjopen-2017-019952
13. Džupina M, Džupinová Z. Dimensions of Csr in Online Communication of Pharmaceutical Companies: A Comparative Study. International Journal of Entrepreneurial Knowledge [Internet]. 2019 Dec [cited 2021 Dec 9];7(2):41–52. Available from: https://proxy.library.carleton.ca/login?url=https://search.ebscohost.com/login.aspx?direct=true&db=bth&AN=140498215&site=ehost-live
14. Yıldırım M, Dinçer MAM. How the Process of the CSR Activities Works on Private Hospitals and Pharmaceutical Firms: Multiple Case Study from Strategic Perspective. J Relig Health [Internet]. 2020 Apr 1 [cited 2021 Dec 13];59(2):961–85. Available from: https://doi.org/10.1007/s10943-018-0653-8
15. Bechoux L, De Vleeschouwer O, Vanheuverzwijn C, Verhegghen F, Detiffe A, Colle F, et al. Conflict of interest policies at Belgian medical faculties: Cross-sectional study indicates little oversight. Grundy Q, editor. PLoS ONE [Internet]. 2021 Feb 10 [cited 2021 Nov 29];16(2):e0245736. Available from: https://dx.plos.org/10.1371/journal.pone.0245736

**Appendix 5**

**Places to find internal documents:**

**27 official websites:**

House of Commons Health Committee (<https://www.ourcommons.ca/Committees/en/HESA> ); the Prescription Medicines Code of Practice Authority (PMCPA, <https://www.pmcpa.org.uk/> ); The Medicines and Healthcare products Regulatory Agency (MHRA) is responsible for enforcing marketing regulations (<https://www.gov.uk/government/organisations/medicines-and-healthcare-products-regulatory-agency> ); US Department of Justice (DOJ) press releases (<https://www.justice.gov/news> ); Del. Court of Chancery Rule (<https://courts.delaware.gov/chancery/> ); FDA ([www.fda.gov](http://www.fda.gov)); Federal Judiciary’s Public Access to Court Electronic Records Service Center (e.g., <https://ecf.mad.uscourts.gov/doc1/09502786849>); Court document ([www.furiousseasons.com](http://www.furiousseasons.com): link does not seem to work; Jayanti, 2021, p. 5); Texas court (<https://www.txcourts.gov/about-texas-courts/>); Canadian Life and Health Insurance Association (CLHIA, <https://www.clhia.ca/>); the Ministry of Health and Medical Education (<http://www.behdasht.gov.ir/> ), Social Security Organization (<http://www.msio.org.ir> ); Iran Health Insurance Organization (<http://tamin.ir> ); Imam Khomeini Relief Committee (<http://ihio.gov.ir/> ); Armed Forces Health Care Insurance (http://mod.ir/); as four main insurance organizations in Iran, the Health Committee of the Parliament Profile (<http://rc.majlis.ir/fa/parliament_commission/health> ); strategic vice president’s site (the Management/Planning Organization; <https://www.mporg.ir> ); Supreme Insurance Council (<http://centinsur.ir/>); Healthy Skepticism (<http://www.healthyskepticism.org/documents/>); Automation of Reports and Consolidated Orders System (ARCOS database, <https://www.deadiversion.usdoj.gov/arcos/index.html> ); American Academy of Dermatology Association (AAD, <https://www.aad.org/>); Agency for Healthcare Research and Quality’s National Guideline Clearinghouse (AHRQ, <https://www.ahrq.gov/gam/index.html>; <https://www.ahrq.gov/prevention/guidelines/archive.html> ); National Health Services of England and Wales (NHS Authorities and Trusts, <https://www.nhs.uk/servicedirectories/pages/nhstrustlisting.aspx>); Freedom of Information Act (FOIA, <https://www.foia.gov/>); US Senate Finance Committee (<https://www.finance.senate.gov/>); Litigation ([www.healthyskepticism.org/documents/PaxilStudy329.php](http://www.healthyskepticism.org/documents/PaxilStudy329.php));

**4 academic websites:**

University of Washington_Biostatistics_Rofecoxib Research Information (<https://www.biostat.washington.edu/research/Rofecoxib>); Oregon University_The Drug Effectiveness Review Project (DERP) (<https://www.ohsu.edu/evidence-based-practice-center/derp-reports>); PLOS Medicine (<https://www.plosmedicine.org/static/ghostwriting.action>); University of California, San Francisco (UCSF)’s Tobacco Documents Bibliography at the Tobacco Control Archives.

**11 databases:**

Merck Archives (https://pharmaphorum.com/tag/merck/); MEDLINE (<https://www.nlm.nih.gov/medline/index.html>); The ***EudraGMDP*** database (<http://eudragmdp.ema.europa.eu/inspections/gmpc/index.do>); The FDA’s online Drug Approval Package (DAP) database (<https://www.fda.gov/drugs/development-approval-process-drugs/drug-approvals-and-databases>); PLoS (<http://www.plosmedicine.org.static/ghostwriting.action>); Drug Information Document Archive (<http://dida.library.ucsf.edu/documents.jsp>); Resources page (dida.library.ucsf.edu/resources.jsp); <https://www.clinicaltrials.gov/>; Medical Directory of Australia (MDA; <https://www.mdaonline.com.au/#:~:text=The%20Medical%20Directory%20of%20Australia,and%20facility%20records%20and%20counting>.); Dollars for Docs database-ProPublica (<https://projects.propublica.org/docdollars/>); Ovid search engine (<https://www.wolterskluwer.com/en/solutions/ovid/ovid-medline-901>).
